# Alternative splicing of transcription factors in plant responses to low temperature stress: mechanisms and functions

**DOI:** 10.1007/s00425-013-1882-4

**Published:** 2013-04-28

**Authors:** Pil Joon Seo, Mi-Jeong Park, Chung-Mo Park

**Affiliations:** 1 Department of Chemistry, Chonbuk National University, Jeonju, 561-756 Korea; 2 Department of Chemistry, Seoul National University, Seoul, 151-742 Korea; 3 Plant Genomics and Breeding Institute, Seoul National University, Seoul, 151-742 Korea

**Keywords:** Alternative splicing, *Arabidopsis*, Cold stress, Peptide interference (PEPi), Small interfering peptide (siPEP), Splicing factor, Transcription factor

## Abstract

Transcription factors play a central role in the gene regulatory networks that mediate various aspects of plant developmental processes and responses to environmental changes. Therefore, their activities are elaborately regulated at multiple steps. In particular, accumulating evidence illustrates that post-transcriptional control of mRNA metabolism is a key molecular scheme that modulates the transcription factor activities in plant responses to temperature fluctuations. Transcription factors have a modular structure consisting of distinct protein domains essential for DNA binding, dimerization, and transcriptional regulation. Alternative splicing produces multiple proteins having different structural domain compositions from a single transcription factor gene. Recent studies have shown that alternative splicing of some transcription factor genes generates small interfering peptides (siPEPs) that negatively regulate the target transcription factors via peptide interference (PEPi), constituting self-regulatory circuits in plant cold stress response. A number of splicing factors, which are involved in RNA binding, splice site selection, and spliceosome assembly, are also affected by temperature fluctuations, supporting the close association of alternative splicing of transcription factors with plant responses to low temperatures. In this review, we summarize recent progress on the temperature-responsive alternative splicing of transcription factors in plants with emphasis on the siPEP-mediated PEPi mechanism.

## Introduction

Transcription factor is a critical component of the gene regulatory networks that mediate virtually all aspects of plant growth and developmental processes. It also plays a central role in plant responses to biotic and abiotic stresses. Therefore, the transcription factor activities are coordinately regulated at various steps to fine-tune signal transduction pathways in diverse cellular signaling networks for optimal growth and survival under given growth conditions (Shinozaki et al. 2003; Yamaguchi-Shinozaki and Shinozaki 2006). Well-established molecular and biochemical mechanisms underlying regulation of transcription factor activities include gene transcriptional regulation, post-transcriptional regulation of RNA metabolism, protein translation, post-translational modifications, and controlled protein turnover (Yun et al. 2008).

Recent studies have shown that post-transcriptional control of RNA metabolism is widespread in plant genomes. For example, over 60 % of intron-containing genes undergo alternative splicing in *Arabidopsis* (Syed et al. 2012). Alternative splicing provides proteome diversity and, thus, expands the repertoire of gene/protein activities in response to developmental and environmental cues (Matlin et al. 2005; Syed et al. 2012). The number of splice variants would be much more than we expected, as we explore more alternatively spliced variants in different cell types, tissues, developmental stages, and environmental conditions (Syed et al. 2012).

While alternative splicing is an important gene regulatory mechanism per se to generate diverse functional proteins, it is also associated with other gene regulatory mechanisms, such as peptide interference (PEPi) that is mediated by small interfering peptides (siPEPs) (Seo et al. 2011b). Dynamic dimer formation is important for the regulatory specificity and functional reliability of transcription factors (Baxevanis and Vinson 1993). Most transcription factors form homodimers and heterodimers to diversify DNA-binding specificities and target selection (Baxevanis and Vinson 1993; Izawa et al. 1993; Vinson et al. 1993). Notably, dimer formation also underlies the dominant-negative regulation of transcription factors, which is mediated by a group of siPEPs (Seo et al. 2011b).

The siPEPs refer to a distinct class of proteins with unique structural organizations and limited sequence similarities to certain members of transcription factors. They have dimerization domains that are required for protein–protein interactions, but lack functional domains, such as those for DNA binding and/or transcriptional regulation (Yun et al. 2008; Seo et al. 2011b). The known siPEPs have no transcriptional regulatory activity. Instead, they are able to interact with target transcription factors via the dimerization domains homologous to those of the target transcription factors. As a result, the siPEPs competitively interfere with functional dimer formation of the transcription factors (Seo et al. 2011b; Staudt and Wenkel 2011) and, thus, the functional mechanism has been designated PEPi. The PEPi is conceptually similar to the RNA interference (RNAi) that is mediated by small interfering RNAs (siRNAs), such as microRNAs (miRNAs), but distinct from the latter in that the former functions at the protein level (Ramachandran and Chen 2008; Staudt and Wenkel 2011; Naqvi et al. 2012). It has been predicted that over 80 siPEPs are encoded in the *Arabidopsis* genome (Seo et al. 2011b), suggesting that the siPEP-mediated PEPi is a widely conserved transcriptional regulatory mechanism in plant genomes.

Interestingly, alternative splicing is closely associated with siPEP biogenesis. At least part of the splice variants of transcription factors apparently lack functional domains required for DNA binding and transcriptional regulation (Seo et al. 2011b, 2012), indicating that they are transcriptionally inactive. However, it can act as siPEP by forming nonfunctional heterodimers with functional transcription factors, establishing a distinct self-regulatory circuit.

Alternative splicing is also associated with the regulation of mRNA stability. Many splice variants contain premature termination codons that are targeted by the nonsense-mediated decay (NMD) mechanism (Kurihara et al. 2009; Rebbapragada and Lykke-Andersen 2009; Palusa and Reddy 2010). It has been predicted that approximately 10–15 % of splice variants are coupled with NMD in *Arabidopsis* (Kalyna et al. 2012), supporting that alternative splicing mediates controlled turnover of gene transcripts.

An interesting observation is that alternative splicing is often responsive to cold stress in plants (Iida et al. 2004; Palusa et al. 2007). A large portion of transcription factor genes undergoes alternative splicing (Barbazuk et al. 2008; Li et al. 2012a; Mastrangelo et al. 2012; Severing et al. 2012). Accordingly, alternative splicing is considered as a way of perceiving temperature fluctuations and modulating transcription factor activity, perhaps by linking gene expression regulation with the PEPi and/or NMD mechanism, in temperature signaling cascades in plants.

This review summarizes temperature-responsive alternative splicing events in plants and point outs their physiological significance in regulating transcription factor activity. We especially focus on the siPEP-mediated PEPi mechanism in association with alternative splicing events of transcription factor genes. We also provide insights into the biological relevance of alternative splicing as a way of self-regulating transcription factor activities in plant responses to low temperature stress that profoundly affects crop productivity in the cool and temperate zones.

## siPEP as a self-regulatory scheme of transcription factors

### Discovery of plant siPEPs

It has been reported that truncated forms of transcription factors, which do not have DNA-binding domains and/or transcriptional regulation domains, play a dominant-negative role in gene expression regulation (Mizukami et al. 1996; Tzeng and Yang 2001). Although this phenomenon has been employed to design synthetic peptides for targeted inactivation of specific transcription factors (Ferrario et al. 2004), it has not been explored whether this mechanism is a general scheme for transcriptional control in living organisms.

The genomic siPEPs in plants have been discovered by Wenkel et al. (2007); Kim et al. (2008). It has been found that a group of small proteins consisting of less than 150 residues, designated LITTLE ZIPPER 1-4 (ZPR1-4), plays a role in shoot apical meristem (SAM) development and leaf polarity determination. For example, the ZPR3 protein consists of 67 residues. It has a protein–protein interaction domain that has a limited sequence similarity to those of class III homeodomain-leucine zipper (HD-ZIP III) transcription factors, such as REVOLUTA (REV), PHABULOSA (PHB), and PHAVOLUTA (PHV). However, the ZPR3 protein does not possess protein domains required for DNA binding and transcriptional activation and, thus, has no ability to regulate gene transcription but interferes with the transcriptional activities of HD-ZIP III transcription factors by forming nonfunctional heterodimers. Consistent with the dominant-negative regulation of the HD-ZIP III proteins by ZPR3, higher-order mutants of the *ZPR* genes are phenotypically similar to the gain-of-function *phb*-*1D* dominant mutant, and the *phb*-*1D* phenotype is compromised in the *phb*-*1D* × *zpr3*-*1D* plants (Kim et al. 2008). Other ZPR proteins, such as ZPR1, ZPR2, and ZPR4, are predicted to function in a similar manner as the ZPR3 protein (unpublished, Kim et al.).

### Genomic siPEPs in *Arabidopsis*

Based on the structural organization of the ZPR proteins and their functional roles as the dominant-negative regulators of HD-ZIP III transcription factors, at least 80 potential siPEPs have been identified in the *Arabidopsis* genome (Seo et al. 2011b). The newly identified siPEPs, although they lack one or more protein domains required for transcription factor activities, belong to various transcription factor families.

The MINI ZINC FINGER (MIF) proteins have been identified as putative zinc finger (ZF) motif-containing transcription factors functioning in diverse growth hormone signaling and flower architecture (Hu and Ma 2006). Later, it has been found that the MIF proteins do not have transcription factor activities themselves but regulate the activities of the ZF-HOMEODOMAIN (ZHD) transcription factors by competitively forming nonfunctional heterodimers (Hong et al. 2011), similar to what have been observed with the ZPR proteins (Kim et al. 2008).

The atypical helix-loop-helix (HLH) protein LONG HYPOCOTYL IN FAR-RED 1 (HFR1) retains the HLH domain, but has defects in the basic DNA-binding domain. It has been shown that the HFR1 protein plays a dominant-negative role in photomorphogenesis (Hornitschek et al. 2009). As inferred form the structural feature of the HFR1 protein, it interacts with the PHYTOCHROME-INTERACTING FACTOR 4 (PIF4) and PIF5 proteins that are responsible for shade avoidance response by binding directly to the G-boxes in shade marker gene promoters (Hornitschek et al. 2009). HFR1 accumulates in the shade and forms non-DNA-binding heterodimers with the PIF transcription factors, thus fine-tuning plant response to the shade.

It is remarkable that the HFR1 protein itself is also targeted by HLH motif-containing KIDARI (KDR) proteins consisting of ~100 residues. The KDR proteins lack protein domains required for transcription factor activities. They interact with HFR1 through the HLH motif and prevent HFR1 from binding to the PIF transcription factors (Hyun and Lee 2006; Hong et al. 2013), providing a double layer of competitive inhibition for the transcriptional control of PIF target genes.

HFR1 also plays a regulatory role in diverse light responses (Duek and Fankhauser 2003; Yang et al. 2005; Zhang et al. 2008). KDR is crucial for both phytochrome A and cryptochrome 1 signaling (Duek and Fankhauser 2003), and thereby the KDR-HFR1 interaction is relevant to blue and far-red light responses. The PRE3/bHLH135/ATBS1/TMO7 non-DNA-binding HLH motif-containing protein is also supposed to play a role in light signaling through interactions with HFR1. The *PRE3/bHLH135/ATBS1/TMO7* gene is transcriptionally regulated by red, far-red, and blue lights, and transgenic plants overexpressing the *PRE3* gene are accordingly hyposensitive to red, far-red, and blue lights (Castelain et al. 2012). PRE3 physically interacts with HFR1 and, thus, it seems that PRE3 represses the HFR1 action like the KDR proteins.

The HLH motif-containing BANQUO1 (BNQ1)/bHLH136, BNQ2/bHLH134, and BNQ3/bHLH161 proteins also interact with HFR1 (Mara et al. 2010). The BNQ proteins are likely to have somewhat distinct physiological roles in comparison to those of KDR and PRE3. It has been reported that the *BNQ1*, *BNQ2*, and *BNQ3* genes are regulated by floral homeotic proteins APETALA3 (AP3) and PISTILLATA (PI) in floral organogenesis (Mara et al. 2010). Altogether, it seems likely that HFR1 serves as an integrator of diverse input signals mediated by the atypical HLH proteins. Therefore, dynamic interactions and competitions among the HLH proteins constitute a web of complex regulatory networks in plant photomorphogenesis and organ development.

A number of additional siPEPs remain to be functionally characterized in plant genomes. Their physiological roles and mechanistic basis are currently unclear in most cases. They would probably regulate the activities of specific transcription factors through competitive inhibition of the target transcription factors in distinct cellular processes and signaling pathways.

## Alternative splicing in plants

Alternative splicing of primary transcripts has evolved to overcome the limited coding capacities of eukaryotic genomes by producing multiple proteins from a single gene and, thus, enhance the transcriptome diversity and proteome plasticity. Recent advances in high-throughput sequencing techniques allow us to explore the extent of alternative splicing events in plants. It has been estimated that over 60 % of intron-containing genes undergo alternative splicing in plants (Marquez et al. 2012; Syed et al. 2012).

Alternative splicing is involved in a wide range of plant growth and developmental processes, such as flowering induction (Eckardt 2002; Slotte et al. 2009) and plant responses to environmental fluctuations and pathogen attacks (Barbazuk et al. 2008), indicating that enhanced diversity of transcriptomes and proteomes is required to cope with plant developmental fitness and environmental adaptation.

Cold-responsive gene regulation and alternative splicing are frequently associated with each other in plants (Iida et al. 2004; Palusa et al. 2007). For instance, wheat *WDREB2* gene, an *Arabidopsis* *DEHYDRATION-RESPONSIVE ELEMENT BINDING PROTEIN 2* (*DREB2*) gene homolog, produces three different transcripts through exon skipping at low temperatures (Egawa et al. 2006). The three transcript isoforms have different accumulation patterns, and the relative ratio of the transcript isoforms is modulated in response to temperature changes. Likewise, rice *DREB2*-type gene, *OsDREB2B*, also undergoes alternative splicing to produce two isoforms, *OsDREB2B1* and *OsDREB2B2* (Matsukura et al. 2010). Accumulation of the isoforms is differentially regulated by temperatures.

Alternative splicing of transcription factor genes is also responsive to high temperatures. For instance, two transcription factor genes, *CIRCADIAN CLOCK-ASSOCIATED 1* (*CCA1*) and *LATE ELONGATED HYPOCOTYL* (*LHY*) that encode MYB domain-containing members, produce differential isoforms at high ambient temperatures. At warm temperatures, whereas the alternative splicing of the *CCA1* gene is enhanced, that of the *LHY* gene is suppressed (Filichkin et al. 2010; James et al. 2012). In addition, the alternative splicing of the *Arabidopsis* *HEAT SHOCK TRANSCRIPTION FACTOR A2* gene is affected under extreme heat conditions (Liu et al. 2013).

## Alternative splicing as a way of producing siPEP

It has been proposed that plant siPEPs are evolutionarily originated from transcription factor proteins by partial duplication of the transcription factor genes and the duplication point is prior to the diversification of flowering plants (Wenkel et al. 2007; Hu et al. 2008). It is notable that partial loss of the transcription factor domains leads to the generation of siPEPs, which are functionally active players in the regulation of transcription factor activities, rather than production of nonfunctional proteins.

**Fig. 1:**
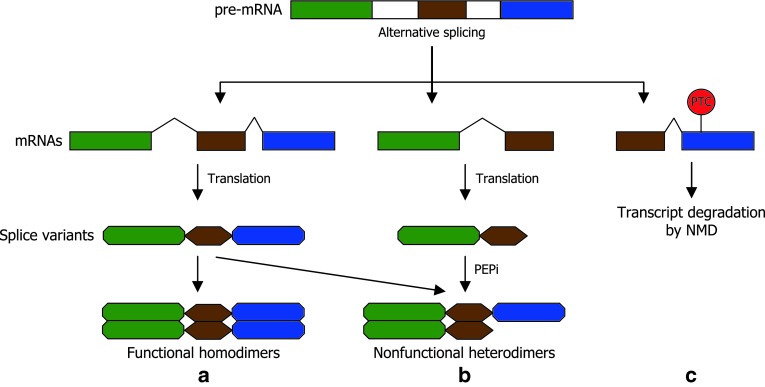
Schematic diagram illustrating the alternative splicing patterns of a transcription factor gene and the fates of splice variants. Transcriptionally functional splice variants form homodimers (**a**). Some splice variants having dimerization domains, but lacking other functional domains may act as siPEPs through peptide interference (PEPi) by competitively inhibiting functional homodimer formation (**b**). Alternative splicing also plays a role in the regulation of mRNA accumulation by producing aberrant transcripts that contain premature stop codon (PTC), which would be degraded by the nonsense-mediated decay (NMD) pathway (**c**)

Alternative selection of 5′ and 3′ splice sites and exon skipping result in the generation of diverse isoforms that have different combinations of functional domains and protein structures (Marquez et al. 2012; Syed et al. 2012). In case of the alternative splicing of transcription factors, some of the splice variants have structures similar to those of genomic siPEPs in that they retain dimerization domains, but lack DNA-binding domains and/or transcriptional regulation domains (Seo et al. 2011a, 2012). The truncated forms would competitively interact with full-size transcription factors to inhibit the activities of the functional forms (Fig. 1). It is noteworthy that a single gene produces both transcripts, one encoding a functional transcription factor and the other encoding a siPEP that negatively regulates the functional form, constituting a self-regulatory loop (Seo et al. 2011b). It is, therefore, envisioned that alternative splicing is a molecular mechanism that modulates protein interaction networks and, thus, the effects of alternative splicing on transcriptional regulation would be much more robust than we expect.

## siPEPs in plant development and physiology

### Starch metabolism

Sugar metabolism is intimately linked with plant adaptation response to environmental stress conditions, such as low temperatures. Soluble sugars accumulate under cold stress conditions and act as compatible osmolytes to reduce ice nucleation in the extracellular regions (Ruelland et al. 2009). Accumulation of starch is also modulated to optimize plant growth under the conditions of limited nutrition availability induced by temperature extremes (Nägele et al. 2011).

Alternative splicing of the *Arabidopsis* *INDETERMINATE DOMAIN 14* (*IDD14*) gene regulates starch metabolism under cold conditions (Seo et al. 2011a). In response to low temperatures, alternative splicing of the *IDD14* gene produces two splice isoforms, *IDD14α* encoding a functional IDD14 transcription factor and *IDD14*β encoding a truncated form, by intron retention. The IDD14β form has defects in the ZF DNA-binding motif, but retains the protein domain required for dimer formation and transcriptional regulation. It interacts with IDD14α to interfere with the formation of IDD14α–IDD14α homodimers Transgenic plants overexpressing the *IDD14*α gene (35S:*IDD14α*) exhibit distinct growth alterations possibly by inducing the *QUA*-*QUINE STARCH* (*QQS*) gene, which is involved in starch degradation (Li et al. 2009). Accordingly, the 35S:*IDD14α* and 35S:*QQS* transgenic plants contain reduced starch contents and exhibit stunted growth phenotypes, whereas the 35S:*IDD14β* transgenic plants contain high starch contents, supporting the dominant-negative role of IDD14β (Seo et al. 2011a). The *IDD14* alternative splicing is induced by cold stress, and accordingly the expression of the *QQS* gene is suppressed under identical conditions.

### Freezing tolerance

At low temperatures, plants trigger a wide array of transcriptional regulatory cascades to induce freezing tolerance. A key regulatory circuit of cold response is composed of C-REPEAT/DEHYDRATION-RESPONSIVE ELEMENT BINDING FACTORS (CBF/DREBs) and its downstream targets *COLD*-*REGULATED* (*COR*) genes and, thus, designated CBF-COR regulon, in higher plants (Badawi et al. 2007; Mao and Chen 2012). The CBF-COR pathway is also associated with the circadian clock (Gilmour et al. 1998; Fowler et al. 2005; Franklin and Whitelam 2007), underscoring the coincidence of endogenous physiology with environmental stimuli.

The core clock components CCA1 and LHY transcriptionally regulate the *CBF* genes by binding directly to the gene promoters (Dong et al. 2011). Consistently, transgenic plants overexpressing the *CCA1* gene exhibit substantially enhanced freezing tolerance, whereas *CCA1*-deficient mutants are sensitive to low temperatures (Dong et al. 2011; Seo et al. 2012).

The *CCA1* gene undergoes alternative splicing, which is suppressed at low temperatures (Park et al. 2012; Seo et al. 2012). The *CCA1* alternative splicing produces two splice variants, *CCA1α* and *CCA1β*. The truncated CCA1β form lacks MYB DNA-binding domain, but has domains responsible for dimerization and transcriptional regulation. It inhibits the DNA binding of CCA1α and LHY by forming non-DNA-binding heterodimers. Consistent with the suppression of the CCA1α activity by CCA1β, the phenotypes of the 35S:*CCA1α* transgenic plants are rescued by *CCA1β* coexpression. At low temperatures, CCA1α is liberated from CCA1β and form CCA1α–CCA1α and LHY-CCA1α dimers, which bind to the *CBF* gene promoters for induction of freezing tolerance (Dong et al. 2011; Seo et al. 2012). In addition to the enhanced freezing tolerance, the 35S:*CCA1β* transgenic plants also exhibit altered rhythmic expression of clock-regulated genes, similar to what observed in *cca1 lhy* double mutants (Seo et al. 2012). These observations indicate that rhythmic expression of cold-responsive genes is also critical for plant adaptation response to low temperatures.

The linkage of the circadian clock with temperature responses via alternative splicing is also observed in other organisms. Alternative splicing of the *FREQUENCY* (*FRQ*) gene links the circadian clock with ambient temperature responses in *Neurospora crassa* (Liu et al. 1997), indicating that alternative splicing provides a critical molecular scheme for the clock to optimize their growth under unfavorable growth conditions. It seems that the alternative splicing-mediated self-regulatory circuits of key transcription factors provide a fine-tuning mechanism of plant adaptation to low temperatures by balancing positive and negative signaling elements.

### Flowering time control

Exposure to a prolonged period of low nonfreezing temperatures promotes flowering in many plant species, which is termed vernalization. The *FLOWERING LOCUS C* (*FLC*) gene is a vital player in the vernalization process in *Arabidopsis* (Kim et al. 2009a; Andrés and Coupland 2012). A MADS-box gene homologous to the *Arabidopsis* *FLC* gene has also been isolated in *Poncirus trifoliata* (Zhang et al. 2009). The *PtFLC* gene is transcriptionally regulated by seasonal temperature fluctuations. It is also regulated post-transcriptionally by alternative splicing by means of exon skipping, resulting in five splice variants. The alternative splicing pattern of the *PtFLC* gene is altered through developmental stages and further influenced by temperature fluctuations (Zhang et al. 2009). The smaller splice variants possibly act as dominant-negative repressors by either competing for DNA binding or forming nonfunctional heterodimers with the functional PtFLC protein (Chen and Coleman 2006; Zhang et al. 2009). Given that the molar ratio of the five splice variants varies through developmental stages and, thus, probably contributes to phase transitions, alternative splicing of the *PtFLC* gene would provide a way of linking temperature signals with endogenous developmental programs in *Poncirus trifoliata*.

### Secondary cell wall biosynthesis

SECONDARY WALL-ASSOCIATED NAC DOMAIN 1 (SND1) transcription factors regulate secondary cell wall biosynthesis by inducing their own genes and *PtrMYB021* gene in *Populus trichocarpa* (Li et al. 2012a). Ectopic expression of the *SND1* genes results in growth retardation as well as ectopic xylogenesis. The *SND1* gene undergoes alternative splicing to produce a truncated *PtrSND1*-*A2* isoform through intron retention. The *SND1*-*A2* isoform suppresses the expression of the *PtrSND1* and *PtrMYB021* genes, suggesting that the splice variant acts as a dominant-negative regulator of the PtrSND1 transcription factors. The PtrSND1-A2 isoform possesses dimerization domain, but lacks DNA-binding and transactivation domains. Accordingly, the PtrSND1-A2 protein forms nonfunctional heterodimers with the functional PtrSND1 transcription factors (Li et al. 2012b).

It is currently unclear whether the alternative splicing of the *PtrSND1* gene is influenced by temperature changes. Secondary cell wall biosynthesis is influenced by cold temperatures (Lefebvre et al. 2011). Therefore, it is possible that alternative splicing of the *PtrSND1* genes serves as a molecular mechanism to optimize vascular development and secondary cell wall biosynthesis under cold conditions.

## Alternative splicing and NMD under cold conditions

Aberrant mRNA transcripts containing premature stop codons are often produced during the transcriptional and post-transcriptional steps. If they are translated, the result would be deleterious gain-of-function or dominant-negative activity of the resulting proteins (Kurihara et al. 2009; Filichkin et al. 2010). Nonsense-mediated mRNA decay (NMD) is a widely conserved eukaryotic pathway that reduces errors in gene expression by eliminating the aberrant mRNAs (Chang et al. 2007; Kurihara et al. 2009). The NMD pathway is mainly mediated by UP-FRAMESHIFT 1 (UPF1), UPF2, and UPF3 proteins in plants (Kim et al. 2009b; Kurihara et al. 2009). It has been found that alternative splicing also regulates the levels of mRNA transcripts by producing mRNA transcripts that contain premature termination codons, which are targeted by NMD (Lareau et al. 2007; Filichkin et al. 2010).

Extensive analyses of large populations of *Arabidopsis* transcripts have shown that the NMD pathway is closely linked with alternative splicing (Palusa and Reddy 2010; Kalyna et al. 2012). The *serine/arginine*-*rich* (*SR*) genes encode RNA-binding proteins that act as splicing regulators. It has been shown that the splice variants of the *SR* genes are targeted by NMD (Palusa and Reddy 2010). Surprisingly, genome-wide high-resolution RT-PCR analyses indicate that approximately 11–18 % of splice variants are degraded by NMD in *Arabidopsis* (Kalyna et al. 2012), supporting the close linkage of alternative splicing with NMD. Although not all transcripts having premature termination codons are targeted by NMD, it seems obvious that the presence of unproductive splice variants can influence the level of functional mRNA transcripts (Fig. 1).

The *LHY* and *PSEUDO RESPONSE REGULATOR 7* (*PRR7*) genes undergo alternative splicing, which is regulated primarily by temperature signals (Filichkin and Mockler 2012; James et al. 2012). Alternative splicing of the *LHY* gene produces a splice variant that is transcriptionally nonfunctional and, thus, is degraded by NMD (Filichkin and Mockler 2012; James et al. 2012). Given that the promoter strength is not affected by temperature changes, it is evident that alternative splicing of the *LHY* gene contributes to reducing the *LHY* gene transcripts (James et al. 2012). It is, therefore, likely that the *LHY* alternative splicing provides an additional layer of regulating the circadian clock function in temperature responses and, thus, compensates the effect of temperature fluctuations on plant development.

## Temperature-responsive splicing factors

A large portion of intron-containing genes in plants undergoes alternative splicing, which is regulated by a set of splicing factors (Syed et al. 2012). Differential regulation of the abundance and activity of splicing factors is critical for the controlled expression of target genes under diverse growth conditions (Palusa et al. 2007). It is notable that a number of splicing factors involved in pre-mRNA splicing are influenced by temperature signals.

The SR splicing factors, a family of splicing regulators with one or two RNA recognition motifs (RRMs) in the N-terminal region and one arg/ser-rich domain in the C-terminal region, are highly conserved in plants (Matlin et al. 2005). They are involved in both constitutive and alternative splicing events and determine the selection of splice sites in a concentration-dependent manner by forming differential spliceosome complexes (Kalyna and Barta 2004; Matlin et al. 2005). The *SR* genes exhibit distinct spatial and temporal expression patterns under fluctuating environmental conditions. The expression of the tomato (*Lycopersicon esculentum*) splicing factor genes, *Le9G8-SR* and *LeSF2-SR1*, is altered dramatically at low temperatures (Fung et al. 2006). In *Arabidopsis*, the expression of *AtSR45a* and *AtSR30* genes is differentially regulated by cold temperatures (Tanabe et al. 2007). Considering that many of the genes encoding splicing factors are influenced by temperature changes and not a few cold-responsive transcription factor genes undergo alternative splicing, alternative splicing is apparently associated with plant responses to temperature changes in many cases.

The splice variants of the SR proteins seem to have distinct functions, further diversifying the regulatory mechanisms in pre-mRNA splicing. At least 95 transcripts are produced from the 15 *SR* genes (Palusa et al. 2007). In addition, their alternative splicing patterns are regulated by diverse environmental cues, such as temperature, light, and growth hormones (Isshiki et al. 2006; Palusa et al. 2007). In particular, heat and cold stress conditions substantially affect the splicing patterns (Palusa et al. 2007). For instance, six splice variants, designated *AtSR45a1*-*a*-*e* and *AtSR45a2*, are generated from the *SR45a* gene by alternative selection of transcriptional initiation sites and alternative splicing of introns. The ratio of the splice variants is altered in plants exposed to low temperatures. The levels of *AtSR45a1*-*a* and *AtSR45a2* transcripts are reduced in response to low temperatures, whereas those of the other four transcripts are unchanged under identical conditions (Tanabe et al. 2007).

The *Arabidopsis* SR1 protein is a plant homolog of the human general/alternative splicing factor SF2/ASF. The *SR1* gene produces five different splice variants through alternative utilization of competing 3′ splice sites and suppression of 5′ splice sites in intron 9 (Lazar and Goodman 2000). The ratio of the *SR1/SR1B* transcripts generated by alternative splicing is under temperature control. The temperature-dependent regulation of the *SR1B/SR1* ratio suggests a role of SR1B in plant adaptation response to ambient temperatures (Lazar and Goodman 2000). In addition, the splicing patterns of a number of other *SR* genes, including *SR1/SR34*, *SR33/SCL33*, *RS31*, *RS40*, and *RSZ32*, are also changed at temperature extremes (Palusa et al. 2007).

Recent reports show that a special type of alternative splicing event occurs at the tandem 3′ splice site. In plants and animals, some of the splice acceptor sites have special consensus sequences that have tandem repeats of the consensus sequence ‘‘NAG’’ (N stands for A, C, G, T) and are, thus, termed ‘‘NAGNAG acceptor’’ sites (Hinzpeter et al. 2010). Alternative splicing at the NAGNAG acceptor sites is widespread in many organisms. Both AG alleles of a NAGNAG acceptor can be chosen by spliceosome and, thus, alternative splicing at the NAGNAG acceptor results in the insertion or deletion of one amino acid. In this regard, alternative selection of the 3′ splice sites diversifies the protein structures and proteome plasticity (Hiller et al. 2004; Schindler et al. 2008). The NAGNAG acceptor sites are frequently found in the *Arabidopsis* genome and remarkably enriched in the genes encoding SR and SR-related proteins (Schindler et al. 2008). Alternative splicing of the *SR* genes at the NAGNAG receptor sites is also responsive to low temperatures (Schindler et al. 2008), further supporting that alternative splicing is intimately associated with temperature responses in plants.

Moreover, a number of splicing factors, other than SR proteins, are also under temperature control. The conserved SNW/Ski-interacting protein (SKIP) domain-containing protein is a splicing factor. It regulates alternative splicing possibly by modulating the recognition or cleavage of the splice donor and acceptor sites (Wang et al. 2012). The SKIP protein interacts with the spliceosomal splicing factor SR45 and is related with alternative splicing of the clock genes, such as *PSEUDORESPONSE REGULATOR7* (*PRR7*) and *PRR9*, in a temperature-dependent manner (Wang et al. 2012). It is likely that the SKIP protein integrates temperature information into the circadian clock oscillators, contributing to maintaining temperature compensation.

The STABILIZED1 (STA1) protein is a pre-mRNA splicing factor that is homologous to the human U5 small ribonucleoprotein-associated 102-kD protein (Lee et al. 2006). The *STA1* gene is induced by cold temperatures to confer freezing tolerance. The *sta1*-*1* mutant has alterations in the alternative splicing patterns of the *COR15A* gene, resulting in hypersensitivity to freezing stress (Lee et al. 2006). The *STA1* gene is crucial for temperature-responsive splicing and the turnover of unstable transcripts to reconcile plant fitness under cold stress conditions.

## Future perspectives

Alternative splicing is widely conserved in eukaryotes. Numerous examples illustrate that temperature-responsive alternative splicing is prevalent in plants and plays a role in a broad spectrum of plant responses to low temperatures by diversifying transcriptomes and proteome plasticity.

Alternative splicing is also linked with diverse gene regulatory mechanisms, such as PEPi that regulates gene transcription by modulating transcription factor activities. Plant siPEPs produced by alternative splicing of transcription factor genes form a self-regulatory circuit, as exemplified by *CCA1* alternative splicing (Park et al. 2012; Seo et al. 2012), establishing an elaborate signaling scheme in plants. It has been estimated that the number of alternatively spliced transcription factor genes is over 330 in *Arabidopsis* and rice (Seo et al. 2011a). Further investigations on the roles of plant siPEPs and underlying molecular mechanisms would unravel the biological relevance of alternative splicing in plant adaptation responses and establish novel functional linkages with other gene regulatory mechanisms, such as chromatin modification (Blencowe 2006).

Alternative splicing is profoundly affected by low temperatures in plants. Therefore, it is perceived as a way of integrating temperature signals into plant development and endogenous cellular physiology. Despite its close association of alternative splicing with temperature signals, just a few responsible splicing factors have been characterized so far. It is also unknown how environmental stress signals affect the activities of splicing factors in most cases. Molecular and biochemical investigations on the splicing factors and phenotypic examinations of plants that are defective in the splicing factors and their target genes would help to understand the molecular mechanisms underlying temperature-responsive alternative splicing of cold-responsive transcription factor genes.

Alternative splicing can be biotechnologically explored as a means of elaborate control of transcription factor activities in crop plants. Engineering of the alternative splicing patterns through mutations in splice sites can be applied for modifying plant development and responses to environmental stresses. Modulations of splicing factor activities would be an alternative approach to precisely control plant functions for improved stress tolerance.

## Abbreviations

CBF: C-repeat binding factor
CCA1: CIRCADIAN CLOCK-ASSOCIATED 1
DREB2: Dehydration-responsive element binding protein 2
HD-ZIP III: Class III homeodomain-leucine zipper
HFR1: LONG HYPOCOTYL IN FAR-RED 1
HLH: Helix-loop-helix
IDD14: INDETERMINATE DOMAIN 14
KDR: KIDARI
LHY: LATE ELONGATED HYPOCOTYL
MIF: MINI ZINC FINGER
NMD: Nonsense-mediated decay
PEPi: Peptide interference
PIF: PHYTOCHROME-INTERACTING FACTOR
PRR: PSEUDORESPONSE REGULATOR
PTC: Premature termination codon
SAM: Shoot apical meristem
siPEP: Small interfering peptide
SR: Serine/arginine-rich
UPF: UP-FRAMESHIFT
ZHD: Zinc finger-homeodomain proteins
ZPR: LITTLE ZIPPER

## References

[CR1] Andrés F, Coupland G (2012). The genetic basis of flowering responses to seasonal cues. Nat Rev Genet.

[CR2] Badawi M, Danyluk J, Boucho B, Houde M, Sarhan F (2007). The *CBF* gene family in hexaploid wheat and its relationship to the phylogenetic complexity of cereal *CBFs*. Mol Genet Genomics.

[CR3] Barbazuk WB, Fu Y, McGinnis KM (2008). Genome-wide analyses of alternative splicing in plants: opportunities and challenges. Genome Res.

[CR4] Baxevanis AD, Vinson CR (1993). Interactions of coiled coils in transcription factors: where is the specificity?. Curr Opin Genet Dev.

[CR5] Blencowe BJ (2006). Alternative splicing: new insights from global analyses. Cell.

[CR6] Castelain M, Le Hir R, Bellini C (2012). The non-DNA-binding bHLH transcription factor PRE3/bHLH135/ATBS1/TMO7 is involved in the regulation of light signaling pathway in *Arabidopsis*. Physiol Plant.

[CR7] Chang YF, Imam JS, Wilkinson MF (2007). The nonsense-mediated decay RNA surveillance pathway. Annu Rev Biochem.

[CR8] Chen K, Coleman G (2006) TypeII MADS-box genes associated with poplar apical bud development and dormancy. Abstract presented at the American Society of Plant Biologists Meeting, Boston MA, USA, pp 5–9 August 2006 (http://abstracts.aspb.org/pb2006/public/P03/P03015.html)

[CR9] Dong MA, Farré EM, Thomashow MF (2011). Circadian clock-associated 1 and late elongated hypocotyl regulate expression of the C-repeat binding factor (CBF) pathway in *Arabidopsis*. Proc Natl Acad Sci USA.

[CR10] Duek PD, Fankhauser C (2003). HFR1, a putative bHLH transcription factor, mediates both phytochrome A and cryptochrome signalling. Plant J.

[CR11] Eckardt NA (2002). Alternative splicing and the control of flowering time. Plant Cell.

[CR12] Egawa C, Kobayashi F, Ishibashi M, Nakamura T, Nakamura C, Takumi S (2006). Differential regulation of transcript accumulation and alternative splicing of a *DREB2* homolog under abiotic stress conditions in common wheat. Genes Genet Syst.

[CR13] Ferrario S, Busscher J, Franken J, Gerats T, Vandenbussche M, Angenent GC, Immink RG (2004). Ectopic expression of the petunia MADS box gene *UNSHAVEN* accelerates flowering and confers leaf-like characteristics to floral organs in a dominant-negative manner. Plant Cell.

[CR14] Filichkin SA, Mockler TC (2012). Unproductive alternative splicing and nonsense mRNAs: a widespread phenomenon among plant circadian clock genes. Biol Direct.

[CR15] Filichkin SA, Priest HD, Givan SA, Shen R, Bryant DW, Fox SE, Wong WK, Mockler TC (2010). Genome-wide mapping of alternative splicing in *Arabidopsis**thaliana*. Genome Res.

[CR16] Fowler SG, Cook D, Thomashow MF (2005). Low temperature induction of *Arabidopsis**CBF1*, *2*, and *3* is gated by the circadian clock. Plant Physiol.

[CR17] Franklin KA, Whitelam GC (2007). Light-quality regulation of freezing tolerance in *Arabidopsis**thaliana*. Nat Genet.

[CR18] Fung RW, Wang CY, Smith DL, Gross KC, Tao Y, Tian M (2006). Characterization of alternative oxidase (AOX) gene expression in response to methyl salicylate and methyl jasmonate pre-treatment and low temperature in tomatoes. J Plant Physiol.

[CR19] Gilmour SJ, Zarka DG, Stockinger EJ, Salazar MP, Houghton JM, Thomashow MF (1998). Low temperature regulation of the *Arabidopsis* CBF family of AP2 transcriptional activators as an early step in cold-induced COR gene expression. Plant J.

[CR20] Hiller M, Huse K, Szafranski K, Jahn N, Hampe J, Schreiber S, Backofen R, Platzer M (2004). Widespread occurrence of alternative splicing at NAGNAG acceptors contributes to proteome plasticity. Nat Genet.

[CR21] Hinzpeter A, Aissat A, Sondo E, Costa C, Arous N, Gameiro C, Martin N, Tarze A, Weiss L, de Becdelièvre A, Costes B, Goossens M, Galietta LJ, Girodon E, Fanen P (2010). Alternative splicing at a NAGNAG acceptor site as a novel phenotype modifier. PLoS Genet.

[CR22] Hong SY, Kim OK, Kim SG, Yang MS, Park CM (2011). Nuclear import and DNA binding of the ZHD5 transcription factor is modulated by a competitive peptide inhibitor in *Arabidopsis*. J Biol Chem.

[CR23] Hong SY, Seo PJ, Ryu JY, Cho SH, Woo JC, Park CM (2013). A competitive peptide inhibitor KIDARI negatively regulates HFR1 by forming nonfunctional heterodimers in *Arabidopsis* photomorphogenesis. Mol Cells.

[CR24] Hornitschek P, Lorrain S, Zoete V, Michielin O, Fankhauser C (2009). Inhibition of the shade avoidance response by formation of non-DNA binding bHLH heterodimers. EMBO J.

[CR25] Hu W, Ma H (2006). Characterization of a novel putative zinc finger gene *MIF1*: involvement in multiple hormonal regulation of *Arabidopsis* development. Plant J.

[CR26] Hu W, dePamphilis CW, Ma H (2008). Phylogenetic analysis of the plant-specific zinc finger-homeobox and mini zinc finger gene families. J Integr Plant Biol.

[CR27] Hyun Y, Lee I (2006). KIDARI, encoding a non-DNA Binding bHLH protein, represses light signal transduction in *Arabidopsis**thaliana*. Plant Mol Biol.

[CR28] Iida K, Seki M, Sakurai T, Satou M, Akiyama K, Toyoda T, Konagaya A, Shinozaki K (2004). Genome-wide analysis of alternative pre-mRNA splicing in *Arabidopsis**thaliana* based on full-length cDNA sequences. Nucleic Acids Res.

[CR29] Isshiki M, Tsumoto A, Shimamoto K (2006). The serine/arginine-rich protein family in rice plays important roles in constitutive and alternative splicing of pre-mRNA. Plant Cell.

[CR30] Izawa T, Foster R, Chua NH (1993). Plant bZIP protein DNA binding specificity. J Mol Biol.

[CR31] James AB, Syed NH, Bordage S, Marshall J, Nimmo GA, Jenkins GI, Herzyk P, Brown JW, Nimmo HG (2012). Alternative splicing mediates responses of the *Arabidopsis* circadian clock to temperature changes. Plant Cell.

[CR32] Kalyna M, Barta A (2004). A plethora of plant serine/arginine-rich proteins: redundancy or evolution of novel gene functions?. Biochem Soc Trans.

[CR33] Kalyna M, Simpson CG, Syed NH, Lewandowska D, Marquez Y, Kusenda B, Marshall J, Fuller J, Cardle L, McNicol J, Dinh HQ, Barta A, Brown JW (2012). Alternative splicing and nonsense-mediated decay modulate expression of important regulatory genes in *Arabidopsis*. Nucleic Acids Res.

[CR34] Kim YS, Kim SG, Lee M, Lee I, Park HY, Seo PJ, Jung JH, Kwon EJ, Suh SW, Paek KH, Park CM (2008). HD-ZIP III activity is modulated by competitive inhibitors via a feedback loop in *Arabidopsis* shoot apical meristem development. Plant Cell.

[CR35] Kim DH, Doyle MR, Sung S, Amasino RM (2009). Vernalization: winter and the timing of flowering in plants. Annu Rev Cell Dev Biol.

[CR36] Kim SH, Koroleva OA, Lewandowska D, Pendle AF, Clark GP, Simpson CG, Shaw PJ, Brown JW (2009). Aberrant mRNA transcripts and the nonsense-mediated decay proteins UPF2 and UPF3 are enriched in the *Arabidopsis* nucleolus. Plant Cell.

[CR37] Kurihara Y, Matsui A, Hanada K, Kawashima M, Ishida J, Morosawa T, Tanaka M, Kaminuma E, Mochizuki Y, Matsushima A, Toyoda T, Shinozaki K, Seki M (2009). Genome-wide suppression of aberrant mRNA-like noncoding RNAs by NMD in *Arabidopsis*. Proc Natl Acad Sci USA.

[CR38] Lareau LF, Brooks AN, Soergel DA, Meng Q, Brenner SE (2007). The coupling of alternative splicing and nonsense-mediated mRNA decay. Adv Exp Med Biol.

[CR39] Lazar G, Goodman HM (2000). The *Arabidopsis* splicing factor SR1 is regulated by alternative splicing. Plant Mol Biol.

[CR40] Lee BH, Kapoor A, Zhu J, Zhu JK (2006). STABILIZED1, a stress-upregulated nuclear protein, is required for pre-mRNA splicing, mRNA turnover, and stress tolerance in *Arabidopsis*. Plant Cell.

[CR41] Lefebvre V, Fortabat MN, Ducamp A, North HM, Maia-Grondard A, Trouverie J, Boursiac Y, Mouille G, Durand-Tardif M (2011). ESKIMO1 disruption in *Arabidopsis* alters vascular tissue and impairs water transport. PLoS One.

[CR42] Li L, Foster CM, Gan Q, Nettleton D, James MG, Myers AM, Wurtele ES (2009). Identification of the novel protein QQS as a component of the starch metabolic network in *Arabidopsis* leaves. Plant J.

[CR43] Li Q, Lin YC, Sun YH, Song J, Chen H, Zhang XH, Sederoff RR, Chiang VL (2012). Splice variant of the SND1 transcription factor is a dominant negative of SND1 members and their regulation in *Populus trichocarpa*. Proc Natl Acad Sci USA.

[CR44] Li Q, Zhang C, Li J, Wang L, Ren Z (2012). Genome-wide identification and characterization of R2R3MYB family in *Cucumis sativus*. PLoS One.

[CR45] Liu Y, Garceau NY, Loros JJ, Dunlap JC (1997). Thermally regulated translational control of FRQ mediates aspects of temperature responses in the neurospora circadian clock. Cell.

[CR46] Liu J, Sun N, Liu M, Liu J, Du B, Wang X, Qi X (2013). An autoregulatory loop controlling *Arabidopsis**HsfA2* expression: role of heat shock-induced alternative splicing. Plant Physiol doi.

[CR47] Mao D, Chen C (2012). Colinearity and similar expression pattern of rice DREB1s reveal their functional conservation in the cold-responsive pathway. PLoS One.

[CR48] Mara CD, Huang T, Irish VF (2010). The *Arabidopsis* floral homeotic proteins APETALA3 and PISTILLATA negatively regulate the *BANQUO* genes implicated in light signaling. Plant Cell.

[CR49] Marquez Y, Brown JW, Simpson C, Barta A, Kalyna M (2012). Transcriptome survey reveals increased complexity of the alternative splicing landscape in *Arabidopsis*. Genome Res.

[CR50] Mastrangelo AM, Marone D, Laidò G, De Leonardis AM, De Vita P (2012). Alternative splicing: enhancing ability to cope with stress via transcriptome plasticity. Plant Sci.

[CR51] Matlin AJ, Clark F, Smith CW (2005). Understanding alternative splicing: towards a cellular code. Nat Rev Mol Cell Biol.

[CR52] Matsukura S, Mizoi J, Yoshida T, Todaka D, Ito Y, Maruyama K, Shinozaki K, Yamaguchi-Shinozaki K (2010). Comprehensive analysis of rice DREB2-type genes that encode transcription factors involved in the expression of abiotic stress-responsive genes. Mol Genet Genomics.

[CR53] Mizukami Y, Huang H, Tudor M, Hu Y, Ma H (1996). Functional domains of the floral regulator AGAMOUS: characterization of the DNA binding domain and analysis of dominant negative mutations. Plant Cell.

[CR54] Nägele T, Kandel BA, Frana S, Meissner M, Heyer AG (2011). A systems biology approach for the analysis of carbohydrate dynamics during acclimation to low temperature in *Arabidopsis**thaliana*. FEBS J.

[CR55] Naqvi AR, Sarwat M, Hasan S, Roychodhury N (2012). Biogenesis, functions and fate of plant microRNAs. J Cell Physiol.

[CR56] Palusa SG, Reddy AS (2010). Extensive coupling of alternative splicing of pre-mRNAs of serine/arginine (SR) genes with nonsense-mediated decay. New Phytol.

[CR57] Palusa SG, Ali GS, Reddy AS (2007). Alternative splicing of pre-mRNAs of *Arabidopsis* serine/arginine-rich proteins: regulation by hormones and stresses. Plant J.

[CR58] Park MJ, Seo PJ, Park CM (2012). *CCA1* alternative splicing as a way of linking the circadian clock to temperature response in *Arabidopsis*. Plant Signal Behav.

[CR59] Ramachandran V, Chen X (2008). Small RNA metabolism in *Arabidopsis*. Trends Plant Sci.

[CR60] Rebbapragada I, Lykke-Andersen J (2009). Execution of nonsense-mediated mRNA decay: what defines a substrate?. Curr Opin Cell Biol.

[CR61] Ruelland E, Vaultier MN, Zachowski A, Hurry V, Kader JC, Delseny M (2009). Cold signalling and cold acclimation in plants. Adv Bot Res.

[CR62] Schindler S, Szafranski K, Hiller M, Ali GS, Palusa SG, Backofen R, Platzer M, Reddy AS (2008). Alternative splicing at NAGNAG acceptors in *Arabidopsis**thaliana* SR and SR-related protein-coding genes. BMC Genomics.

[CR63] Seo PJ, Kim MJ, Ryu JY, Jeong EY, Park CM (2011). Two splice variants of the IDD14 transcription factor competitively form nonfunctional heterodimers, which may regulate starch metabolism. Nat Commun.

[CR64] Seo PJ, Hong SY, Kim SG, Park CM (2011). Competitive inhibition of transcription factors by small interfering peptides. Trends Plant Sci.

[CR65] Seo PJ, Park MJ, Lim MH, Kim SG, Lee M, Baldwin IT, Park CM (2012). A self-regulatory circuit of *CIRCADIAN CLOCK-ASSOCIATED1* underlies the circadian clock regulation of temperature responses in *Arabidopsis*. Plant Cell.

[CR66] Severing EI, van Dijk AD, Morabito G, Busscher-Lange J, Immink RG, van Ham RC (2012). Predicting the impact of alternative splicing on plant MADS domain protein function. PLoS One.

[CR67] Shinozaki K, Yamaguchi-Shinozaki K, Seki M (2003). Regulatory network of gene expression in the drought and cold stress responses. Curr Opin Plant Biol.

[CR68] Slotte T, Huang HR, Holm K, Ceplitis A, Onge KS, Chen J, Lagercrantz U, Lascoux M (2009). Splicing variation at a *FLOWERING LOCUS C* homeolog is associated with flowering time variation in the tetraploid *Capsella bursa-pastoris*. Genetics.

[CR69] Staudt AC, Wenkel S (2011). Regulation of protein function by ‘microProteins’. EMBO Rep.

[CR70] Syed NH, Kalyna M, Marquez Y, Barta A, Brown JW (2012). Alternative splicing in plants–coming of age. Trends Plant Sci.

[CR71] Tanabe N, Yoshimura K, Kimura A, Yabuta Y, Shigeoka S (2007). Differential expression of alternatively spliced mRNAs of *Arabidopsis* SR protein homologs, atSR30 and atSR45a, in response to environmental stress. Plant Cell Physiol.

[CR72] Tzeng TY, Yang CH (2001). A MADS box gene from lily (*Lilium Longiflorum*) is sufficient to generate dominant negative mutation by interacting with PISTILLATA (PI) in *Arabidopsis**thaliana*. Plant Cell Physiol.

[CR73] Vinson CR, Hai T, Boyd SM (1993). Dimerization specificity of the leucine zipper-containing bZIP motif on DNA binding: prediction and rational design. Genes Dev.

[CR74] Wang X, Wu F, Xie Q, Wang H, Wang Y, Yue Y, Gahura O, Ma S, Liu L, Cao Y, Jiao Y, Puta F, McClung CR, Xu X, Ma L (2012). SKIP is a component of the spliceosome linking alternative splicing and the circadian clock in *Arabidopsis*. Plant Cell.

[CR75] Wenkel S, Emery J, Hou BH, Evans MM, Barton MK (2007). A feedback regulatory module formed by LITTLE ZIPPER and HD-ZIPIII genes. Plant Cell.

[CR76] Yamaguchi-Shinozaki K, Shinozaki K (2006). Transcriptional regulatory networks in cellular responses and tolerance to dehydration and cold stresses. Annu Rev Plant Biol.

[CR77] Yang J, Lin R, Sullivan J, Hoecker U, Liu B, Xu L, Deng XW, Wang H (2005). Light regulates COP1-mediated degradation of HFR1, a transcription factor essential for light signaling in *Arabidopsis*. Plant Cell.

[CR78] Yun J, Kim SG, Hong S, Park CM (2008). Small interfering peptides as a novel way of transcriptional control. Plant Signal Behav.

[CR79] Zhang XN, Wu Y, Tobias JW, Brunk BP, Deitzer GF, Liu D (2008). HFR1 is crucial for transcriptome regulation in the cryptochrome 1-mediated early response to blue light in *Arabidopsis**thaliana*. PLoS One.

[CR80] Zhang JZ, Li ZM, Mei L, Yao JL, Hu CG (2009). PtFLC homolog from trifoliate orange (*Poncirus trifoliata*) is regulated by alternative splicing and experiences seasonal fluctuation in expression level. Planta.

